# Acute respiratory infections in hospitalized children in Vientiane, Lao PDR – the importance of Respiratory Syncytial Virus

**DOI:** 10.1038/s41598-017-09006-6

**Published:** 2017-08-24

**Authors:** Van Hoan Nguyen, Audrey Dubot-Pérès, Fiona M. Russell, David A. B. Dance, Keoudomphone Vilivong, Souphatsone Phommachan, Chanthaphone Syladeth, Jana Lai, Ruth Lim, Melinda Morpeth, Mayfong Mayxay, Paul N. Newton, Hervé Richet, Xavier De Lamballerie

**Affiliations:** 1 UMR “Emergence des Pathologies Virales” (EPV: Aix-Marseille university - IRD190 - Inserm 1207 - EHESP), Marseille, France; 2 Institut hospitalo-universitaire Méditerranée infection, APHM Public Hospitals of Marseille, Marseille, France; 3 Department of Infectious Diseases, Hai Phong University of Medicine and Pharmacy, Hai Phong, Vietnam; 4 Lao-Oxford-Mahosot Hospital-Wellcome Trust Research Unit (LOMWRU), Microbiology Laboratory, Vientiane, Lao PDR; 5 Centre for Tropical Medicine and Global Health, Nuffield Department of Clinical Medicine, Old Road Campus, University of Oxford, Oxford, United Kingdom; 6 Dept. of Paediatrics, The University of Melbourne, Melbourne, Australia; 7 Pneumococcal Research Group, Murdoch Childrens Research Institute, The Royal Children’s Hospital, Melbourne, Australia; 8 Faculty of Infectious and Tropical Diseases, London School of Hygiene and Tropical Medicine, London, UK; 9 National Centre for Epidemiology & Population Health, Australian National University, Canberra, Australia; 10 The Royal Children’s Hospital, Melbourne, Australia; 11 Faculty of Postgraduate Studies, University of Health Sciences, Vientiane, Lao PDR, Vientiane, Laos

## Abstract

The *Human respiratory syncytial virus* (RSV) is one of the most important viral pathogens, causing epidemics of acute respiratory infection (ARI), especially bronchiolitis and pneumonia, in children worldwide. To investigate the RSV burden in Laos, we conducted a one-year study in children <5 years old admitted to Mahosot Hospital, Vientiane Capital, to describe clinical and epidemiological characteristics and predictive factors for severity of RSV-associated ARI. Pooled nasal and throat swabs were tested using multiplex real-time PCR for 33 respiratory pathogens (FTD^®^ kit). A total of 383 patients were included, 277 (72.3%) of whom presented with pneumonia. 377 (98.4%) patients were positive for at least one microorganism, of which RSV was the most common virus (41.0%), with a peak observed between June and September, corresponding to the rainy season. Most RSV inpatients had pneumonia (84.1%), of whom 35% had severe pneumonia. Children <3-months old were a high-risk group for severe pneumonia, independently of RSV infection. Our study suggests that RSV infection is frequent in Laos and commonly associated with pneumonia in hospitalized young children. Further investigations are required to provide a better overall view of the Lao nationwide epidemiology and public health burden of RSV infection over time.

## Introduction

In 2012, the World Health Organization (WHO) launched the Battle against Respiratory Viruses (BRaVe) initiative in response to increasing evidence that viruses play an important role in acute respiratory infections (ARI). WHO emphasized the need to prioritize research to gain a better understanding of the epidemiology, pathogenesis, prevention and clinical management of respiratory virus infections across different populations and resource settings^1^.

Indeed, ARI are still the leading killer of children less than five years of age, accounting for ~17% of the 10.4 million deaths worldwide^2^. Respiratory viruses account for approximately half of all cases of community-acquired pneumonia and more than 90% of bronchiolitis cases in children in low- and middle-income countries^3–5^. Although a significant number of studies on respiratory viral infections have been published, there is still a poor evidence-base for understanding the impact of these infections and of potential pharmacological interventions^6^.

*Human respiratory syncytial virus* (RSV) is one of the most important respiratory viral pathogens in young children, frequently causing bronchiolitis and pneumonia^7^. Nair *et al*. estimated that, in 2005, 33.8 million new episodes of RSV-associated Acute Lower Respiratory tract Infection (ALRI) occurred worldwide in children younger than 5 years old, with at least 3.4 million episodes necessitating hospitalization, and 66,000 to 199,000 deaths, 99% of which occurred in developing countries^5^. However, the importance of RSV-associated ALRI remains underestimated in many developing countries, where virology data are often not available and possibly hidden by bacterial pathogens, especially *Streptococcus pneumoniae* and *Haemophilus influenzae* ^8^.

The WHO Product Development for Vaccines Advisory Committee highlighted the priority for global healthcare of developing an RSV vaccine^9^. Several candidates have been evaluated in clinical trials. However, no approved vaccine for RSV is yet available^10, 11^. For successful prevention of RSV infection, a prophylactic vaccine approach would be the logistically easiest approach if a safe and effective RSV vaccine was to become available, but epidemiological data are needed to plan implementation of a vaccine campaign.

In the Lao People’s Democratic Republic (Lao PDR, Laos), respiratory infections are poorly characterized with only limited data available. The first influenza surveillance system established in Lao PDR with laboratory diagnosis included 526 patients of all ages, including 160 (30.4%) children <5 years old, with influenza-like illness from January 2007 to December 2008. At least one respiratory virus was detected in 54.6% of the samples; *Influenzavirus* A/B was the most common virus detected and only 9 (3.5%) patients were RSV-positive^12^. Between August 2009 and October 2010, samples from 292 patients of all ages admitted to two Lao hospitals (in Vientiane Capital and Northern Luang Prabang Province) with ALRI were collected for respiratory pathogen investigations^13^. *Human rhinovirus* and RSV were the most frequent viruses detected, in 35% and 26% of patients, respectively. RSV was found in all age groups but was more frequent in children under 5 years old. There is thus a crucial need to collect information to estimate the burden of RSV in Lao PDR to guide public health policy.

The aim of this study was to describe the clinical features, epidemiology and pathogens in hospitalized children - less than 5 years old with ARI - at a central hospital in Vientiane Capital, and to identify predictive factors for severity.

## Methods

This prospective study was conducted in Vientiane Capital, Lao PDR, from December 2013 to December 2014 at Mahosot Hospital, a 365-bed primary-tertiary hospital with approximately 2,000 admissions per month. Patients were included in the three wards where children with acute respiratory illness would be admitted: general paediatric, paediatric infectious diseases, and paediatric ICU.

### Patient inclusion

All children under 5 years old admitted to these three wards were enrolled according to the following criteria: written parental consent to participating in the study; time from onset of symptoms <14 days, fever (axillary temperature >38.0 °C) or history of fever, and at least one respiratory symptom (dyspnea, cough, rhinitis) or abnormal pulmonary auscultation on physical examination.

### Data collection

Demographic, epidemiologic, clinical data and measurements were collected using a questionnaire by physicians from our research team, by interviews and consulting medical charts. The same physicians performed physical examination and sample collection.

### Case definition

Pneumonia and severe pneumonia were defined according to WHO criteria^14^. Children who presented with cough or difficulty breathing and had fast breathing (aged 2–11 months: ≥50 breaths/minute, aged 1–4 years: ≥40 breaths/minute) or chest indrawing, were classified as having pneumonia. Children who presented with cough or difficulty breathing and had at least one of the following criteria were classified as severe pneumonia: oxygen saturation <90%, while breathing room air, or central cyanosis; severe respiratory distress; signs of pneumonia with a general danger sign (inability to breastfeed or drink, lethargy or reduced level of consciousness, convulsions, vomiting). Children <2 months old who presented with cough or difficulty breathing and fast breathing (≥60 breaths/min) were classified as severe pneumonia.

### Pneumococcal vaccination status

Pneumococcal vaccination (PCV) was launched in Lao PDR in November 2013. Therefore, in our study, only children <2 years old could have had access to PCV13 vaccination (Pneumococcal Conjugate Vaccination against 13 pneumococcal serotypes). Children <2 years old were defined as ‘PCV13 received’ if they had received, as reported on their vaccination booklet, at least two doses of vaccine for children <1 year old (one dose is unlikely to be effective against carriage or disease in those <1 year old) and at least one dose for children between 1 to 2 years old.

### Sample collection and laboratory assays

Nasal and throat swab specimens were collected from all children enrolled and placed separately in 2 × 1mL Virocult vials (viral transport medium, Sigma Virocult^®^, MWE). Virocult vials were taken to the laboratory within 2 hours at ambient temperature. The swab was immediately unloaded by strongly squeezing the swab inside the medium, against the bottom of the tube for 30 seconds then outside of the medium against the inner wall of the tube, to release the maximum amount of liquid from the swab. Media were aliquoted and stored at −80 °C before performing the laboratory assays. 100 µL of nasal swab and 100 µL of throat swab media were pooled together for each patient and extracted using the Cador Pathogen 96 QIAcube HT kit (Qiagen) following the manufacturer’s instructions, with an elution of 90 µL. Extracts were tested for 33 pathogens using the FTD^®^ respiratory pathogens 33 kit (Fast-track Diagnostics) which consists of multiplexed Taqman real-time PCRs for the detection of: *Influenzavirus* A; *Influenzavirus* B; *Influenzavirus* C; *Human rhinovirus*; *Human coronavirus* NL63, 229E, OC43, HKU1; *Human parainfluenza virus* 1, 2, 3, 4; *Human metapneumovirus* A and B; *Bocavirus*; *Human respiratory syncytial virus* A and B; *Human adenovirus*; *Human enterovirus*; *Human parechovirus*; *Cytomegalovirus*; *Mycoplasma pneumoniae*; *Chlamydia pneumoniae*; *Staphylococcus aureus*; *Streptococcus pneumoniae*; *Haemophilus influenzae*; *Haemophilus influenzae* type b; *Pneumocystis jirovecii*; *Bordetella* spp.; *Moraxella catarrhalis*; *Klebsiella pneumoniae*; *Legionella* spp.; *Salmonella* spp. The real-time PCR assays were considered as positive if the cycle threshold (Ct) value was <35.

### Statistical analysis

For patient descriptions, we compared RSV-positive with RSV-negative patients within two groups of patients: all ARI patients and ARI patients with pneumonia. For the assessment of predictive factors for severity, patients with severe outcomes were compared with patients with no severe outcome within 6 groups of patients: all ARI patients, RSV-positive ARI patients, RSV-negative ARI patients, and those same three groups in patients less than 2-year old. Death, stay in ICU, oxygen use and severe pneumoniae were considered as indicators for severity. However, the mortality rate was too low to be analyzed and the analyses of ICU stay and oxygen use did not provide any added-value, so ultimately severe pneumonia alone was analyzed as an indicator of severe outcome. Data were double entered into an Access database (Microsoft Corporation). Statistical analysis was performed using Statistical Package for the Social Sciences version 23.0 for Windows (SPSS Inc., Chicago, IL, USA). For comparison of categorical data, the Pearson Chi-square (χ^2^) test and Fisher’s exact test were used as appropriate. The ANOVA test was applied to compare continuous variables. We fitted a binary logistic regression model, including a stepwise selection procedure, to assess predictive factors for severity. The level of significance was set at p < 0.05.

### Ethics

The study was approved by the National Ethics Committee for Health Research, Ministry of Health, Lao PDR, and the Oxford Tropical Research Ethics Committee. Methods were carried out in accordance with the relevant guidelines and regulations. Informed signed consent was obtained from their legal guardians for all children included in this study.

## Results

### ARI patient characteristics

**Figure 1:**
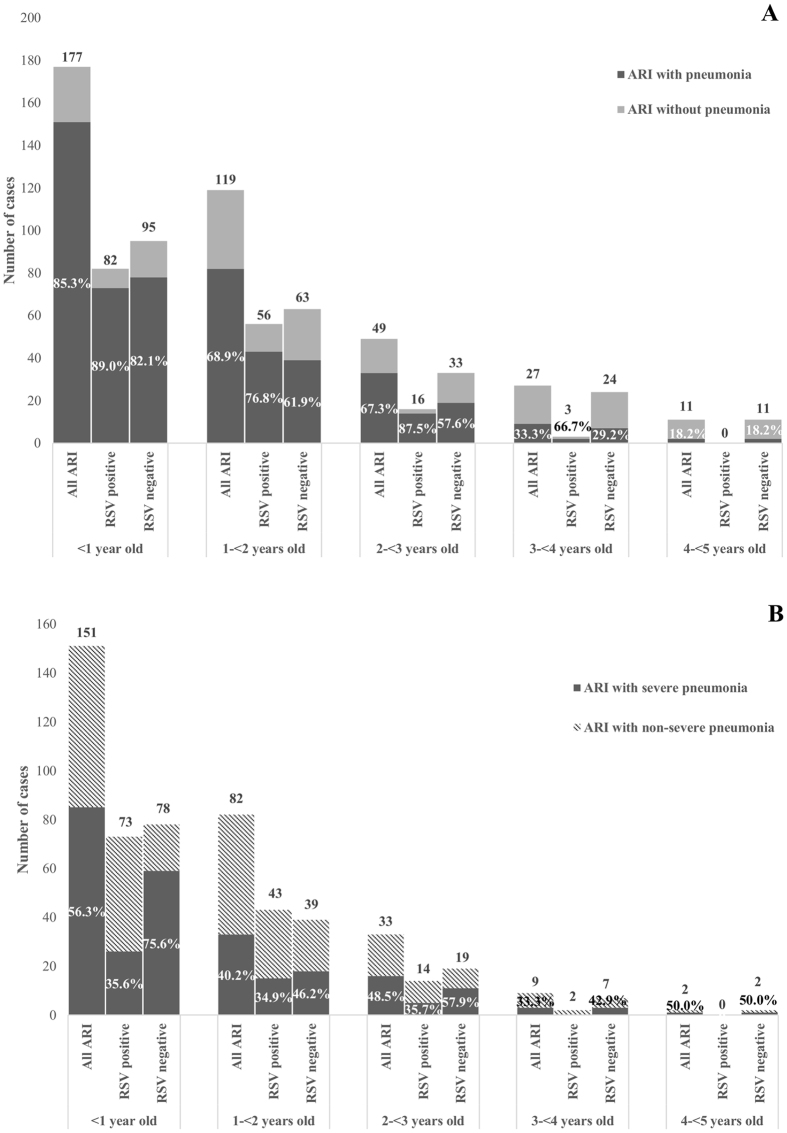
Distribution of ARI patients, with or without pneumonia, by age groups according to RSV status. (**A**) Proportion of pneumonia among ARI patients. (**B**) Proportion of severe pneumonia among ARI patients with pneumonia.

To cover the potential seasonal variability of different agents, we included patients over a full year from 19 December 2013 to 31 December 2014. 407 patients were included. Twenty-four patients were excluded because of missing questionnaires or specimens. Thus, 383 patients with ARI were included in the final analysis, with a sex ratio (M:F) of 1.3. The median age was 13 months (IQR 5–22 months) (Table 1). Two hundred and seventy-seven (72.3%) patients had pneumonia. The age group with the highest proportion of pneumonia was that including those younger than one year (85.3%) (Fig. 1). Among patients <2 years old, 143 (53.4%) had received PCV13. Vaccinated children were significantly more frequent in the less than 1-year-old age group (61.3%) than in the 1 to <2-year-old age group (42.5%, p = 0.002, Figure S1). During the one-year study period, 5 in hospital deaths were reported, with three patients being <1 year old. All fatalities were diagnosed with severe pneumonia according to the WHO criteria.

**Table 1:** Baseline characteristics of ARI patients (n = 383).

Characteristics	n (%)
**Demographic**	
Age (months), median (IQR)	13 (5–22)
Age groups	
*Less than 1-month-old*	14 (3.7)
*1 to less than 3-month-old*	42 (11.0)
*3 –month-old to less than 1-year-old*	121 (31.6)
*1 to less than 2-year-old*	119 (31.1)
*2 to less than 3-year-old*	49 (12.8)
*3 to less than 4-year-old*	27 (7.0)
*4 to less than 5-year-old*	11 (2.9)
Gender (Male)	219 (57.2)
Birth weight (g), median (IQR)	3,000 (2,700–3,400)
*Low birth weight* ^347¥^	46 (13.3)
Wards	
*ICU*	58 (15.1)
*Paediatric Infectious Disease*	58 (15.1)
*General paediatric*	267 (69.7)
Season (Wet season*)	256 (66.8)
PCV13 received^268 ♯^	143 (53.4)
**Clinical presentation**	
Duration of illness prior to hospitalization (day), median (IQR)	3 (2–5)
Fever documented at enrolment^381^	235 (61.7)
Cough^381^	368 (96.6)
Difficulty breathing^375^	275 (73.3)
Coryza^380^	339 (89.2)
Diarrhea^372^	155 (41.7)
Conjunctival suffusion^361^	12 (3.3)
Convulsions^378^	40 (10.6)
Rigors^328^	34 (10.4)
Vomiting^374^	208 (55.6)
**Physical examination**	
Chest indrawing^378^	216 (57.1)
Nasal flaring^371^	86 (23.2)
Lymphadenopathy^359^	21 (5.8)
Abnormal pulmonary auscultation^366^	258 (70.5)
Tachypnea^364^	171 (47.0)
Oxygen saturation <90% in room air^342^	47 (13.7)
Rash^366^	24 (6.6)
Respiratory distress^367^	108 (29.4)
Central cyanosis^372^	46 (12.4)
Wheeze^369^	113 (30.6)
Stridor^365^	16 (4.4)
Grunting^369^	18 (4.9)
Inability to drink^368^	45 (12.2)
Prostration or lethargy^368^	27 (7.3)
**Outcomes**	
Pneumonia	277 (72.3)
Severe pneumonia	138 (36.0)
Length of stay ≤ 5 days^376^	305 (81.1)
Supplementary oxygen used^378^	69 (18.3)
Death	5 (1.3)
**Detection of:**	
*S. pneumoniae*	213 (55.6)
*H. influenzae*	204 (53.3)
*Influenzavirus*	29 (7.6)
*Human parainfluenza virus*	37 (9.7)
*Human metapneumovirus*	6 (1.6)
*Human adenovirus*	28 (7.3)
*Human rhinovirus*	70 (18.3)

Data in the table are number of patients and percentages, except for a few variables for which the unit is specified.

Because of missing values, superscript numbers indicate the number of patients for whom data were available.

Low birth weight: defined by World Health Organisation as weight at birth less than 2,500 g^31^.

Fever: defined as body temperature ≥38 °C per axilla^32^.

Children less than 2 years old were defined as ‘PCV13 received’ if they had received: at least two doses of vaccine for children less than 1 year old or at least one dose of vaccine for children between 1 to 2 years old.

Wet season: from May to October.

### Pathogen detection

Among the 383 children included, 377 (98.4%) were positive by real-time PCR for at least one pathogen. 321 (83.8%) of patients included were positive for respiratory viruses, RSV was detected in 157 (41.0%). One hundred and forty-seven (93.6%) RSV-positive samples were also positive for at least one other microorganism, 77 (49.0%) for other respiratory viruses, and 144 (91.7%) for bacteria.

Of the RSV-positive ARI children, a complete list of viruses co-detected is provided as supplemental data (Table S1). Co-detection with viruses known to be frequent causes of acute respiratory infection were observed: *Human rhinovirus* in 12 (7.6%), *Human adenovirus* in 6 (3.8%), *Human parainfluenza virus* in 3 (1.9%), *Influenzavirus* in 1 (0.6%). *Human metapneumovirus* was not detected in any RSV-positive patients. The main bacterial species co-detected with RSV were *S. pneumoniae* and *H. influenzae* in 98 (62.4%) and 84 (53.5%), respectively.

### Characteristics of ARI patients positive for RSV

**Figure 2:**
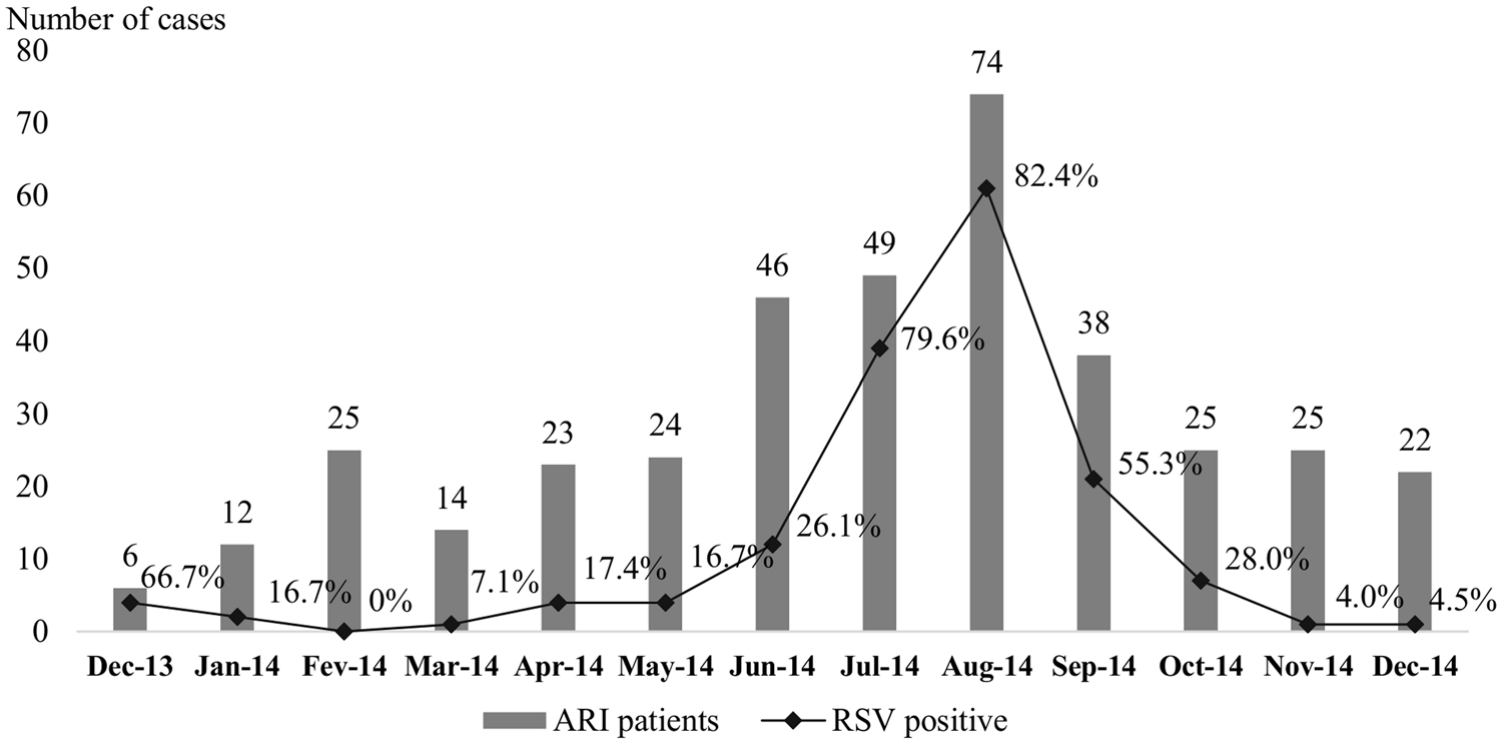
Monthly detection rate of RSV in ARI patients.

The demographic and clinical features of the 157 RSV patients are shown in Table 2. Over half of the RSV patients (52.2%) were less than one year old, 87.9% were less than 2 years old and all were less than 4 years old (Fig. 1). Figure 2 shows the monthly frequency of RSV patients over the one-year study period. One hundred and forty-four (97.1%) of the RSV patients were detected between May and October, with a clear peak between June and September, corresponding to 84.7% of annual RSV cases. Two RSV patients (1.3%) died.

**Table 2:** Characteristics of ARI patients positive for RSV in comparison to ARI patients negative for RSV.

Characteristics	RSV-positive n (%)	RSV-negative n (%)	OR (95% CI)	p-value
Number of patients	157 (41.0)	226 (59.0)		
**Demographics**				
Age (months), median (IQR)	11 (4–18)	14 (6–27)		
Age groups				
*Less than 1-month-old*	10 (6.4)	4 (1.8)	reference	
*1 to less than 3-month-old*	16 (10.2)	26 (11.5)	0.2 (0.06–0.9)	0.061
*3 –month-old to less than 1-year-old*	56 (35.7)	65 (28.8)	0.3 (0.1–1.1)	0.09
*1 to less than 2-year-old*	56 (35.7)	63 (27.9)	0.3 (0.1–1.2)	0.09
*2 to less than 3-year-old*	16 (10.2)	33 (14.6)	0.2 (0.05–0.7)	0.013
*3 to less than 4-year-old*	3 (1.9)	24 (10.6)	0.05 (0.01–0.3)	<0.001
*4 to less than 5-year-old*	0 (0.0)	11 (4.9)	NA	<0.001
Gender (Male)	94 (59.9)	125 (55.3)	1.2 (0.7–1.8)	0.217
Birth weight (g), median (IQR)	3,100 (2,700–3,400)	3,000 (2,700–3,300)		
*Low birth weight* ^347¥^	17 (12.1)	29 (14.0)	0.8 (0.4–1.6)	0.369
Wards				
*ICU*	27 (17.2)	31 (13.7)	1.3 (0.7–2.2)	0.214
*Paediatric Infectious Disease*	17 (10.8)	41 (18.1)	0.5 (0.3–1.0)	0.033
*General Paediatric*	113 (72.0)	154 (68.1)	1.2 (0.7–1.8)	0.246
Season (Wet season*)	144 (97.1)	112 (49.6)	11.2 (6.0–21.0)	<0.001
PVC 13 received^268♯^	68 (57.1)	75 (50.3)	1.3 (0.8–2.0)	0.268
**Clinical presentation**				
Duration of illness prior to hospitalization (day), median (IQR*)*	4 (3–5)	3 (2–5)		
Fever documented at enrolment^381^	94 (60.6)	141 (62.4)	0.9 (0.6–1.4)	0.406
Cough^381^	155 (99.4)	213 (94.7)	8.7 (1.1–67.9)	0.010
Difficulty breathing^375^	134 (87.0)	141 (63.8)	3.8 (2.2–6.5)	<0.001
Coryza^380^	149 (95.5)	190 (84.8)	3.8 (1.6–8.8)	0.001
Diarrhea^372^	75 (48.4)	80 (36.9)	1.6 (1.1–2.4)	0.017
Conjunctival suffusion^361^	1 (0.7)	11 (5.1)	0.1 (0.02–0.9)	0.016
Convulsions^378^	6 (3.9)	34 (15.2)	0.2 (0.09–0.5)	<0.001
Rigors^328^	6 (4.7)	28 (13.9)	0.3 (0.1–0.8)	0.005
Vomiting^374^	93 (60.8)	115 (52.0)	1.4 (0.9–2.2)	0.058
**Physical examination**				
Chest indrawing^378^	115 (74.2)	101 (45.3)	3.5 (2.2–5.4)	<0.001
Nasal flaring^371^	43 (28.7)	43 (19.5)	1.7 (1.02–2.7)	0.027
Lymphadenopathy^359^	2 (1.3)	19 (9.0)	0.1 (0.03–0.6	0.001
Abnormal pulmonary auscultation^366^	137 (91.3)	121 (56.0)	8.3 (4.4–15.5)	<0.001
Tachypnea^364^	89 (58.9)	82 (38.55)	2.3 (1.5–3.5)	<0.001
Oxygen saturation<90% in room air^342^	17 (11.5)	30 (15.5)	0.7 (0.4–1.3)	0.184
Rashes^366^	8 (5.4)	16 (7.4)	0.7 (0.3–1.7)	0.296
Respiratory distress^367^	48 (32.7)	60 (27.3)	1.3 (0.8–2.0)	0.161
Central cyanosis^372^	20 (13.2)	26 (11.8)	1.1 (0.6–2.1)	0.393
Wheeze^369^	51 (33.6)	62 (28.6)	1.3 (0.8–1.9)	0.182
Stridor^365^	3 (2.0)	13 (6.0)	0.3 (0.09–1.1)	0.053
Grunting^369^	5 (3.4)	13 (5.9)	0.6 (0.2–1.6)	0.193
Inability to drink^368^	17 (11.3)	28 (12.9)	0.9 (0.5–1.6)	0.380
Prostration or lethargy^368^	9 (6.0)	18 (8.3)	0.7 (0.3–1.6)	0.263
**Outcomes**				
Pneumonia	132 (84.1)	145 (64.2)	2.9 (1.8–4.9)	<0.001
Severe pneumonia	46 (29.3)	92 (40.7)	0.6 (0.4–0.9)	0.014
Length of stay ≤ 5 days ^376^	132 (85.2)	173 (78.3)	1.6 (0.9–2.8)	0.060
Supplementary oxygen used^378^	29 (18.7)	40 (17.9)	1.1 (0.6–1.8)	0.476
Death	2 (1.3)	3 (1.4)	0.9 (0.2–5.8)	0.666
**Detection of:**				
*S. pneumoniae*	98 (62.4)	115 (50.9)	1.6 (1.1–2.4)	0.016
*H. influenzae*	84 (53.5)	120 (53.1)	1.0 (0.7–1.5)	0.510
*Influenzavirus*	1 (0.6)	28 (12.4)	0.05 (0.01–0.3)	<0.001
*Human parainfluenza virus*	3 (1.9)	34 (15.0)	0.1 (0.03–0.4)	<0.001
*Human metapneumovirus*	0 (0.0)	6 (2.7)	NA	0.041
*Human adenovirus*	6 (3.8)	22 (9.7)	0.4 (0.1–0.9)	0.021
*Human rhinovirus*	12 (7.6)	58 (25.7)	0.2 (0.1–0.5)	<0.001

Data in the table are number of patients and percentages, except for a few variables for which the unit is specified.

Because of missing values, superscript numbers indicate the number of patients for whom data were available.

Low birth weight: defined by World Health Organisation as weight at birth less than 2,500 g^31^.

Fever: defined as body temperature ≥38 °C per axilla^32^.

Children less than 2 years old were defined as ‘PCV13 received’ if they had received: at least two doses of vaccine for children less than 1 year old or at least one dose of vaccine for children between 1 to 2 years old.

Wet season: from May to October.

One hundred and thirty-two (84.1%) RSV patients had pneumonia, which was significantly more frequent than in RSV-negative patients (64.2%, p < 0.001) (Table 2). When stratified by age group (Fig. 1A), no significant difference (p > 0.05) was observed, except for the 2 to <3-year-old group (87.5% with pneumonia in RSV-positive, 57.6% with pneumonia in RSV-negative, p = 0.035).

Severe pneumonia was negatively associated with RSV infection (40.7% of severe pneumonia in RSV-negative patients and 29.3% in RSV-positive patients, p = 0.014) (Table 2). When considering RSV patients with pneumonia, the proportion of severe pneumonia (35%) was similar in all age groups (Fig. 1B), whereas in RSV-negative patients with pneumonia, severe pneumonia was associated with the one-year-old group (75.6%) in comparison to the other age groups (42.9–57.9%, p > 0.005, Fig. 1B).

Coryza (95.5%), difficulty breathing (87.0%) chest indrawing (74.2%), and abnormal pulmonary auscultation (91.3%) were significantly associated with RSV infection in ARI patients as well as in ARI patients with pneumonia (Table 2, Supplemental data Table S2).

*Influenzavirus*, *Human parainfluenza virus*, *Human metapneumovirus*, *Human adenovirus*, and *Human rhinovirus* were significantly more frequent in RSV-negative patients than in RSV-positive patients (p < 0.05), whereas, *S. pneumoniae* detection was significantly associated with RSV infection (p = 0.016) (Table 2). When stratified by age group, this association was significant only for patients aged between 1 and <2 years old (p = 0.026, Supplemental data Figure S2). There was no apparent association between *H. influenzae* and RSV infection status.

Among the <2 year olds, half of the RSV patients (57.1%) had received PCV13, with a similar proportion observed in RSV-negative patients (50.3%, p = 0.268).

### Predictive factors for severity

Predictive factors for severe pneumonia for all ARI patients, RSV-positive and RSV-negative patients were assessed using univariate followed by multivariable analyses (Supplemental Tables S3 to S5). Given that most of RSV patients were younger than 2 years old, the analyses were also performed within just this subgroup of patients (Supplemental data S6 to S8).

Age less than 3 months was the only factor that was independently associated with severe pneumonia in all patient groups (p ≤ 0.009).

Detection of *Human adenovirus* was associated with severe pneumonia in all ARI and RSV-negative patient groups but not in the RSV-positive patient group.

Presentation during the wet season (from May to October) was independently associated with severe pneumonia only in the all RSV-negative patient group (p = 0.024), and borderline in all ARI patient (p = 0.050).

In patients <2 years old, male gender was negatively associated with severe pneumonia in RSV-positive patients (p = 0.020), and ‘PCV13 received’ was protective against severe pneumonia in RSV-negative patients (p = 0.001).

## Discussion

We report for the first time the epidemiological and clinical features of RSV infection in ARI patients in Laos. RSV was found to be the most frequent virus detected (41.0% of recruited ARI patients). In our study, 47.7% of children with pneumonia were infected by RSV. This finding is consistent with others studies performed in several countries in the region^15–17^.

RSV outbreaks are known to vary from year to year and according to geographical patterns. A peak of RSV infection (84.7%) was observed from June to September (Fig. 2), corresponding to the rainy season in Laos. This is consistent with the association of RSV infection with the rainy season reported in studies elsewhere in the tropics^15, 18, 19^. The explanation for this consistent association is unclear. It could be directly related to climatic factors, such as increased humidity, or reflect changes in social behaviour which may enhance transmission^8, 19^. Recently, Paynter reported that the wet conditions of the tropical rainy season may encourage contact transmission of this virus by increasing the amount of virus on surfaces and by increasing virus survival in droplets on surfaces^20^. However, other patterns of RSV seasonality in tropical areas have been described. There were peaks of RSV-associated hospitalization observed during cool periods from 2011 to 2014 in Bangladesh^21^. Substantial differences in geography and climate are present in Laos, especially between the south and the north of the country, and therefore additional data, collected over several years and from different areas, are required to fully describe the seasonality of RSV infection in Laos.

Among RSV-positive patients, 84.1% had pneumonia, of which 35% had severe pneumonia, with no significant differences between age groups. A similar finding was reported in two studies of hospitalized patients in Thailand, in which RSV was the viral pathogen most commonly associated with pneumonia in children under 5 years old^15, 16^. In Laos, pneumonia was less frequent in RSV-negative than in RSV-positive patients. However, severe pneumonia was more frequent in RSV-negative patients (40.7%) than in RSV-positive patients (29.3%), especially in the children <1 year old, in whom the proportion with severe pneumonia was significantly higher in RSV-negative patients (62.1%, p < 0.001). The majority (87.9%) of RSV-positive patients were less than 2 years old. This is similar to the pattern of RSV epidemiology in children worldwide^22–24^ and makes this age group an important target for interventions such as vaccination, which might significantly reduce the incidence of pneumonia.

PCV13 vaccination was launched in Vientiane in November 2013, just one month before the beginning of our study. Therefore, it is highly unlikely to see the impact of PCV on pneumonia with such a small cohort and only 1 year post vaccine introduction.

A low mortality was observed in both RSV-positive (1.3%) and RSV-negative (1.4%) patients. All five deaths were in the group with severe pneumonia, and four cases occurred in children less than one year old. This mortality is lower than the estimate of 3% to 9% associated with RSV infection reported by Nair *et al*.^25^. Beliefs and practices when death is approaching are different in each country. In Southeast Asian culture, relatives usually wish children that they expect to die, to die at home^26^. This belief leads to discharge just before death, and hence may be a reason for the low apparent mortality rate in this study.

*S. pneumoniae* is also an important cause of respiratory tract infections. A synergistic interaction between RSV and *S. pneumoniae* has been proposed, either through the viruses rendering the epithelium more susceptible to bacterial colonization and inducing changes in immune function that might be favourable to bacterial invasion, or by bacterial colonization enhancing respiratory virus infections^27, 28^. In this study, *S. pneumoniae* was detected in a high proportion of RSV-positive patients (62.4%), but the association of *S. pneumoniae* with RSV infection was significant only in the 1 to <2-year-old age group (p = 0.026).

Co-detection of RSV and *Human rhinovirus*, *Human adenovirus*, *Influenzavirus*, or *Human parainfluenza virus* was infrequent, and *Human metapneumovirus* was not detected in any RSV-positive patients whereas they were found in RSV-negative patients. This finding suggests mutual exclusion of respiratory viruses for concomitant infections, as proposed by Pascalis *et al*. for *Influenzavirus* ^29^.

In a multivariable logistic model to analyse the predictive factors for severity, age <3 months was independently associated with severe pneumonia in ARI patients, independent of RSV infection. This is consistent with other studies, confirming the vulnerability of this age group to severe respiratory infections. This is probably due to the immaturity of the young infant’s immune system, with low levels of cellular immunity and sub-optimal antibody responses^30^. The low overall mortality observed in our study did not allow us to investigate predictive factors for mortality.

Limitations of the current study were that it was conducted only on hospitalized children, in a single hospital, and over a short period of time. Therefore, more extensive studies on RSV infections in Laos are required, over a longer period of time and at locations representative of Lao geographical diversity and socio-environmental conditions. A case-control study, as well as collection of comprehensive data on patient’s environments, would enable the identification of risk factors for RSV infection. A case-control study would also permit investigating asymptomatic microorganism carriage. Moreover, it would be interesting to look at the RSV types, to analyse the differences in demographic, clinical features and severity of illness between RSV-A and RSV-B.

Our study provides a baseline snapshot of RSV epidemiology in children hospitalized with ARI in Vientiane Capital. RSV is an important public health issue in Laos, and possibly underestimated. This study shows that RSV is likely to be a major cause of ARI and pneumonia in young hospitalized children in Laos. There is an urgent need to develop more extensive studies in Laos, both in hospitals but also in the community, in order to have better estimates of burden of RSV over the country and over time, in order to implement effective public health intervention programs.

## Electronic supplementary material

Supplemental information
