# Supplementary material for: Acute respiratory infections in hospitalized children in Vientiane, Lao PDR – the importance of Respiratory Syncytial Virus
Source: Sci Rep. 2017 Aug 24;7:9318. doi: 10.1038/s41598-017-09006-6 (PMC5571090; doi:10.1038/s41598-017-09006-6)
Supplement: Supplementary file 1 — Supplemental information [file 41598_2017_9006_MOESM1_ESM.pdf]

Supplementary information

Acute respiratory infections in hospitalized children in Vientiane, Lao PDR – the importance of Respiratory Syncytial Virus.

Authors: Van Hoan Nguyen, Audrey Dubot-Pérès, Fiona M Russell, David AB Dance[4], Keoudomphone Vilivong, Souphatsone Phommachan, Chanthaphone Syladeth, Jana Lai, Ruth Lim, Melinda Morpeth, Mayfong Mayxay, Paul N Newton, Hervé Richet, Xavier De Lamballerie.

Legend

Figure S1: Distribution of PCV13 status by age groups and RSV PCR results in ARI patients.

Figure S2: Distribution of *S. pneumoniae* detected by age groups and RSV PCR results in ARI patients.

Table S1: Co-detection of RSV with other respiratory viruses.

Table S2: Demographic and clinical features of ARI patients with pneumonia.

Table S3: Predictive factors for severe pneumonia in all ARI patients.

Table S4: Predictive factors for severe pneumonia in RSV positive ARI patients.

Table S5: Predictive factors for severe pneumonia in RSV negative ARI patients.

Table S6: Predictive factors for severe pneumonia in ARI patients less than 2-year-old.

Table S7: Predictive factors for severe pneumonia in RSV positive ARI patients less than 2-year-old.

Table S8: Predictive factors for severe pneumonia in RSV negative ARI patients less than 2-year-old.

Figure S1 :

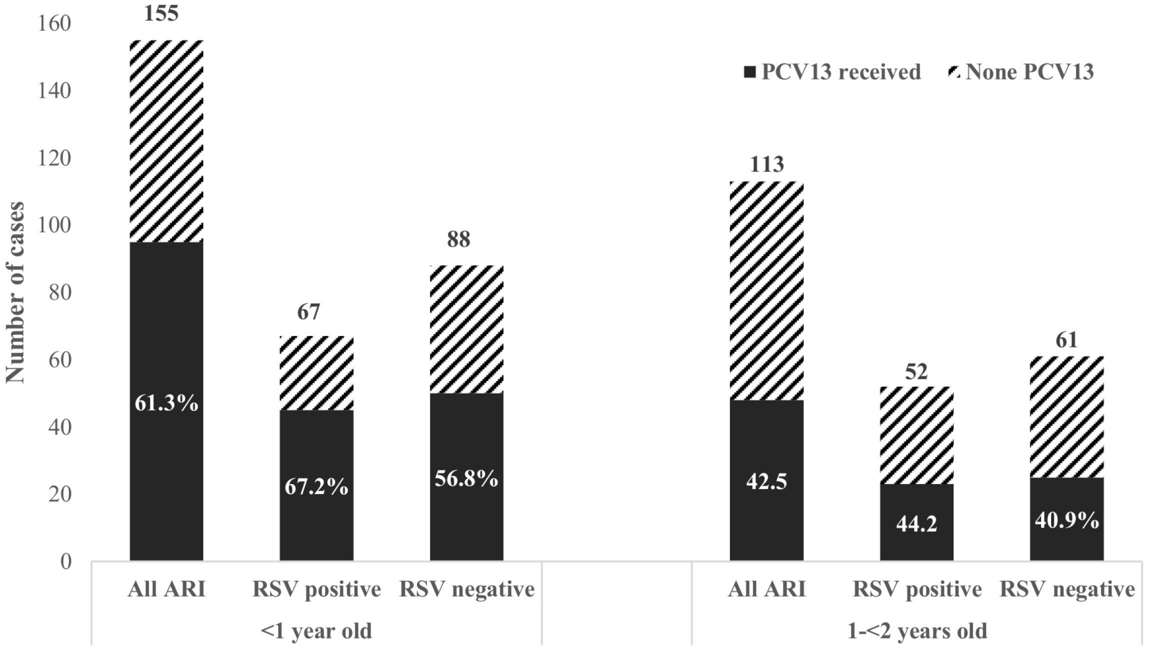
155
160
140
120
100
80
60
40
20
0
Number of cases
PCV13 received
None PCV13
113
88
67
61
52
61.3%
67.2%
56.8%
42.5
44.2
40.9%
All ARI
RSV positive
RSV negative
<1 year old
All ARI
RSV positive
RSV negative
1-<2 years old

Figure S2 :

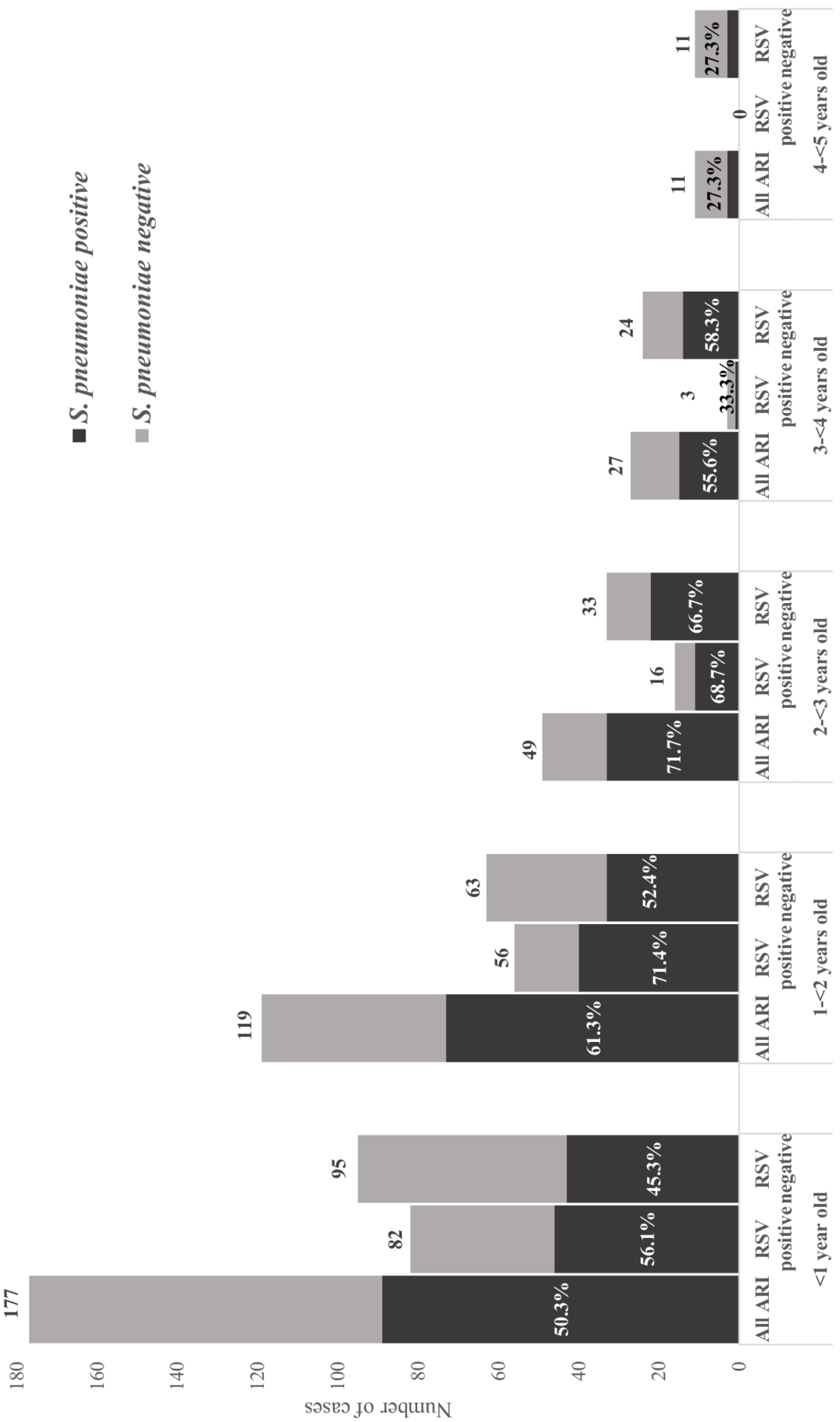

S. pneumoniae positive
S. pneumoniae negative
Number of cases
0
20
40
60
80
100
120
140
160
180
All ARI
RSV positive
RSV negative
<1 year old
1-<2 years old
2-<3 years old
3-<4 years old
4-<5 years old
177
50.3%
82
56.1%
95
45.3%
119
61.3%
56
71.4%
63
52.4%
49
71.7%
16
68.7%
33
66.7%
27
55.6%
3
33.3%
24
58.3%
11
27.3%
0
11
27.3%

Table S1: Co-detection of RSV with other respiratory viruses

Number of patients	Co-detection			
49	RSV	*Cytomegalovirus*		
4	RSV	*Human rhinovirus*		
3	RSV	*Human coronavirus*		
2	RSV	*Human adenovirus*		
1	RSV	*Bocavirus*		
1	RSV	*Parainfluenza virus*		
4	RSV	*Cytomegalovirus*	*Human rhinovirus*	
3	RSV	*Cytomegalovirus*	*Human adenovirus*	
2	RSV	*Cytomegalovirus*	*Parainfluenza virus*	
2	RSV	*Cytomegalovirus*	*Bocavirus*	
1	RSV	*Cytomegalovirus*	*Human coronavirus*	
1	RSV	*Cytomegalovirus*	*Bocavirus*	*Influenza virus*
1	RSV	*Cytomegalovirus*	*Bocavirus*	*Human rhinovirus*
1	RSV	*Cytomegalovirus*	*Human rhinovirus*	*Enterovirus/Parechovirus*
1	RSV	*Cytomegalovirus*	*Human rhinovirus*	*Human adenovirus*
1	RSV	*Human rhinovirus*	*Human coronavirus*	*Enterovirus/Parechovirus*

Table S2: Demographic and clinical features of ARI patients with pneumonia.

**Characteristics**	**Pneumonia RSV positive n (%)**	**Pneumonia RSV negative n (%)**	**OR (95% IC)**	**p-value**
**Patient number**	**132 (47.7)**	**145 (52.3)**		
**Demographic features**				
Age (months), median (IQR)	9 (3-16)	8 (3-16)		
Age groups				
Less than 1-month-old	10 (7.6)	4 (2.8)	reference	
1 to less than 3-month-old	15 (11.4)	23 (15.9)	0.3 (0.06-0.9)	0.061
3 –month-old to less than 1-year-old	48 (36.4)	51 (35.2)	0.3 (0.1-1.3)	0.15
1 to less than 2-year-old	43 (32.6)	39 (26.9)	0.4 (0.1-1.5)	0.249
2 to less than 3-year-old	14 (10.6)	19 (13.1)	0.3 (0.07-1.1)	0.110
3 to less than 4-year-old	2 (1.5)	7 (4.8)	**0.1 (0.01-0.8)**	**0.03**
4 to less than 5-year-old	0 (0.0)	2 (1.4)	NA	0.125
Male	78 (59.1)	83 (57.2)	1.1 (0.7-1.7)	0.425
Birth weight (gr), Median (IQR)	3000 (2600-3400)	3000 (2500-3300)		
Low birth weight[249¥]	17 (14.5)	24 (18.2)	0.8 (0.4-1.5)	0.274
Wards				
ICU	26 (19.7)	30 (20.7)	0.9 (0.5-1.7)	0.478
Paediatric Infectious disease	7 (5.3)	14 (9.7)	0.5 (0.2-1.3)	0.127
Paediatric general	99 (75.0)	101 (69.7)	1.3 (0.8-2.2)	0.196
Season (Wet season) *	120 (90.9)	66 (45.5)	11.9 (6.1-23.6)	<0.001
PVC 13 received[210#]	59 (57.8)	42 (38.9)	2.2 (1.2-3.4)	0.004
**Clinical presentation**				
Duration of illness prior to hospitalization (day)[247] *Median (IQR)*	3 (3-5)	3 (2-5)		
Mean (SD)	3.9 (1.7)	3.6 (2.3)		0.162
Fever documented at inclusion[276]	76 (58.0)	83 (52.2)	1.0 (0.6-1.7)	0.497
Cough[277]	131 (99.2)	142 (97.9)	2.8 (0.3-26.9)	0.347
Sputum[263]	103 (81.1)	91 (66.9)	2.1 (1.2-3.7)	0.006
Runny nose[276]	126 (95.5)	123 (85.4)	3.5 (1.4-9.2)	0.004
Difficulty of breathing[274]	122 (93.1)	114 (79.7)	3.4 (1.7-7.6)	0.001
Conjunctival suffusion[258]	1 (0.8)	6 (4.4)	0.2 (0.02-1.5)	0.076
Diarrhea[270]	63 (47.7)	52 (37.7)	1.5 (0.9-2.5)	0.061
Nausea[116]	4 (6.6)	12 (21.8)	0.3 (0.08-0.8)	0.017
Convulsions[273]	6 (4.6)	32 (22.5)	0.2 (0.07-0.4)	<0.001
Rigors[237]	5 (4.6)	17 (13.2)	0.3 (0.1-0.9)	0.019
Vomiting[271]	76 (58.5)	72 (51.1)	1.3 (0.8-2.2)	0.136
**Physical examination**				
Chest indrawing[274]	115 (87.8)	100 (69.9)	3.1 (1.6-5.8)	<0.001
Abnormal pulmonary auscultation[265]	121 (96.0)	102 (73.4)	8.8 (3.3-23.2)	<0.001
Lymphadenopathy[256]	1 (0.8)	7 (5.3)	0.1 (0.02-1.2)	0.038
Grunting[265]	5 (4.0)	13 (9.3)	0.4 (0.1-1.2)	0.070
Nasal flaring[267]	42 (33.1)	41 (29.3)	1.2 (0.7-2.0)	0.296
Tachypnea[271]	89 (67.9)	81 (57.9)	1.5 (0.9-2.5)	0.056
Oxygen Saturation <90%[260]	17 (13.4)	29 (21.8)	0.5 (0.3-1.1)	0.053
Rashes[262]	6 (4.8)	8 (5.8)	0.8 (0.3-2.4)	0.462
Respiratory distress[263]	47 (38.2)	56 (40.0)	0.9 (0.6-1.5)	0.433

Cyanosis[268]	20 (15.7)	26 (18.4)	0.8 (0.4-1.6)	0.338
Wheeze[267]	49 (38.3)	54 (38.8)	0.9 (0.6-1.6)	0.512
Stridor[262]	3 (2.4)	10 (7.4)	0.3 (0.08-1.1)	0.056
Inability to drink[267]	17 (13.2)	27 (19.6)	0.6 (0.3-1.2)	0.107
Prostration or lethargy[265]	9 (7.1)	18 (13.0)	0.5 (0.2-1.2)	0.080
**Outcomes**				
Severe pneumonia[277]	46 (34.8)	92 (63.4)	0.3 (0.2-0.5)	<0.001
Length of stay ≤5 day[274]	108 (82.4)	104 (72.7)	0.6 (0.3-1.0)	0.037
Oxygen used[275]	29 (22.1)	38 (26.4)	0.8 (0.5-1.4)	0.249
Death[270]	2 (1.6)	3 (2.1)	1.4 (0.2-8.4)	0.542
**Coinfection with:**				
S. pneumoniae	84 (63.6)	77 (53.1)	1.5 (0.9-2.5)	0.049
H. influenzae	72 (54.5)	76 (52.4)	1.1 (0.7-1.7)	0.407
Influenza virus	1 (0.8)	14 (9.7)	0.07 (0.01-0.6)	0.001
Parainfluenza virus	3 (2.3)	28 (19.3)	0.1 (0.03-0.3)	<0.001
Human metapneumovirus	0 (0.0)	3 (2.1)	NA	0.142
Human adenovirus	4 (3.0)	14 (9.7)	0.3 (0.09-0.9)	0.021
Human rhinovirus	12 (9.1)	46 (31.7)	0.2 (0.1-0.4)	<0.001

Data in the table are number of patient and percentages, except for a few variables for which the unit is specified.
¥ Number of patients for whom data were available
Low birth weight: defined by World Health Organisation as weight at birth less than 2500g [31].
Fever: defined as body temperature ≥38°C per axilla [32].
♯ PCV13 received: patients less than 2-year-old who received at least one dose of PCV13.
* Wet season: from May to October

Table S3: Predictive factors for severe pneumonia in all ARI patients.

Factors	Univariate analysis				Multivariable analysis	
	With severe pneumonia n=138 (%)	Without severe pneumonia n=245 (%)	OR (95% IC)	p-value	OR (95% IC)	p-value
Age groups						
Less than 1-month-old	9 (6.5)	5 (2.0)				
1 to less than 3-month-old	29 (21.0)	13 (5.3)				
3 –month-old to less than 1-year-old	47 (34.1)	74 (30.2)				
1 to less than 2-year-old	33 (23.9)	86 (35.1)				
2 to less than 3-year-old	16 (11.6)	33 (13.5)				
3 to less than 4-year-old	3 (2.2)	24 (9.8)				
4 to less than 5-year-old	1 (0.7)	10 (4.1)				
Age less than 3-month-old*	38 (27.5)	18 (7.3)	**4.8 (2.6-8.8)**	**<0.001**	**6.0 (2.9-12.3)**	**<0.001**
Gender, Male	74 (53.6)	145 (59.2)	0.8 (0.5-1.2)	0.171	0.8 (0.5-1.3)	0.328
Birth weight (gr), Mean (SD)	3018.6 (621.3)	2973.4 (570.7)		0.495		
Low birth weight	16 (13.1)	30 (13.3)	0.9 (0.5-1.9)	0.548	0.7 (0.3-1.5)	0.347
Season (Wet season)	80 (58.0)	176 (71.8)	**0.5 (0.3-0.8)**	**0.004**	0.6 (0.3-1.0)	0.050
Duration of illness prior to hospitalization (day), mean (SD)	3.6 (2.1)	4.0 (2.1)				
Duration of illness ≤5 days	112 (81.8)	197 (81.1)	1.0 (0.6-1.8)	0.493	1.2 (0.6-2.1)	0.639
Temperature, mean, (SD)	38.0 (0.9)	38.1 (0.9)		0.436		
Fever documented at inclusion	79 (57.2)	156 (64.2)	0.7 (0.5-1.1)	0.109	0.9 (0.5-1.5)	0.666
**Detection of:**						
S. pneumoniae	78 (56.5)	135 (55.1)	1.1 (0.7-1.6)	0.436	1.2 (0.7-1.9)	0.518
Human respiratory syncytial virus	46 (33.3)	111 (45.3)	**0.6 (0.4-0.9)**	**0.014**	0.9 (0.5-1.7)	0.781
Influenza virus	10 (7.2)	19 (7.8)	0.9 (0.4-2.1)	0.515	1.4 (0.5-3.4)	0.517
Parainfluenza virus	17 (12.3)	20 (8.2)	1.6 (0.8-3.1)	0.127	0.6 (0.3-1.3)	0.188
Human adenovirus	14 (10.1)	14 (5.7)	1.9 (0.9-4.0)	0.083	**3.1 (1.3-7.3)**	**0.012**
Human rhinovirus	32 (23.2)	38 (15.5)	1.6 (0.9-2.8)	**0.043**	1.8 (0.9-3.4)	0.080

* When looking at distribution by age groups, the difference between the proportion of patients with and without severe pneumonia was more important for the age groups below 3-month-old, so for univariate and multivariate analyses we considered the factor “less than 3-month-old”. Some factors were excluded: birth weight that is correlated to low birth weight, duration of illness that is correlated to duration of illness ≤5 days, temperature that is correlated to fever documented at inclusion.

Table S4: Predictive factors for severe pneumonia in RSV positive patients.

Factors	Univariate analysis				Multivariable analysis	
	Severe pneumonia	Not severe pneumonia	OR (95% IC)	p-value	OR (95% IC)	p-value
Age groups						
Less than 1-month-old	5 (10.9)	5 (4.5)				
1 to less than 3-month-old	9 (19.6)	7 (6.3)				
3 –month-old to less than 1-year-old	12 (26.1)	44 (39.6)				
1 to less than 2-year-old	15 (32.6)	41 (36.9)				
2 to less than 3-year-old	5 (10.9)	11 (9.9)				
3 to less than 4-year-old	0 (0.0)	3 (2.7)				
Age < 3 months*	14 (30.4)	12 (10.8)	**3.6 (1.5-8.6)**	**0.004**	**4.5 (1.6-13.0)**	**0.005**
Gender, Male	23 (50.0)	71 (64.0)	0.6 (0.3-1.1)	0.075	0.4 (0.2-1.0)	0.056
Birth weight (gr), Mean (SD)	2973.3 (652.8)	3007.0 (570.8)		0.761		
Low birth weight	4 (10.0)	13 (13.0)	0.7 (0.2-2.4)	0.431	0.7 (0.2-2.6)	0.614
Season (Wet season)	42 (93.1)	102 (91.9)	0.9 (0.3-3.2)	0.563	1.0 (0.2-4.1)	0.981
Duration of illness prior to hospitalization (day), mean (SD)	4.0 (1.9)	4.0 (1.8)		0.887		
Duration of illness ≤5 days	35 (76.1)	91 (82.7)	0.7 (0.3-1.5)	0.228	0.6 (0.2-1.6)	0.310
Temperature, mean, (SD)	38.1 (0.9)	38.0 (0.8)		0.543		
Fever documented at inclusion	27 (58.7)	67 (61.5)	0.9 (0.4-1.8)	0.441	1.2 (0.5-2.4)	0.710
**Detection of:**						
S. pneumoniae	30 (65.2)	68 (61.3)	1.2 (0.6-2.4)	0.390	1.1 (0.5-2.5)	0.896
Influenza virus	0 (0.0)	1 (0.9)	NA	0.707	NA	
Parainfluenza virus	2 (4.3)	1 (0.9)	5.0 (0.4-56.5)	0.206	0.2 (0.01-2.2)	0.173
Human adenovirus	1 (2.2)	5 (4.5)	0.5 (0.05-4.1)	0.432	0.4 (0.03-4.0)	0.407
Human rhinovirus	4 (8.7)	8 (7.2)	1.2 (0.4-4.3)	0.488	2.5 (0.6-10.8)	0.237

* When looking at distribution by age groups, the difference between the proportion of patients with and without severe pneumonia was more important for the age groups below 3-month-old, so for univariate and multivariate analyses we considered the factor “less than 3-month-old”. Some factors were excluded: birth weight that is correlated to low birth weight, duration of illness that is correlated to duration of illness ≤5 days, temperature that is correlated to fever documented at inclusion.

Table S5: Predictive factors for severe pneumonia in RSV negative patients.

Factors	Univariate analysis				Multivariable analysis	
	**Severe pneumonia**	**Not severe pneumonia**	**OR (95% IC)**	**p-value**	**OR (95% IC)**	**p-value**
Age groups						
Less than 1-month-old	4 (4.3)	0 (0.0)				
1 to less than 3-month-old	20 (21.7)	6 (4.5)				
3 –month-old to less than 1-year-old	35 (38.0)	30 (22.4)				
1 to less than 2-year-old	18 (19.6)	45 (33.6)				
2 to less than 3-year-old	11 (12.0)	22 (16.4)				
3 to less than 4-year-old	3 (3.3)	21 (15.7)				
4 to less than 5-year-old	1 (1.1)	10 (7.5)				
Age < 3 months*	24 (26.1)	6 (4.5)	**7.5 (2.9-19.3)**	**<0.001**	**12.3 (3.9-38.5)**	**<0.001**
Gender, Male	51 (55.4)	74 (55.2)	1.0 (0.6-1.7)	0.542	1.1 (0.6-2.0)	0.817
Birth weight (gr), Mean (SD)	3041.2 (607.8)	2946.6 (571.6)		0.258		
Low birth weight	12 (14.6)	17 (13.6)	1.1 (0.5-2.4)	0.494	0.5 (0.2-1.4)	0.208
Season (Wet season)	38 (41.3)	74 (55.2)	**0.6 (0.3-0.9)**	**0.027**	**0.5 (0.3-0.9)**	**0.024**
Duration of illness prior to hospitalization (day), mean (SD)	3.5 (2.2)	3.9 (2.4)		0.184		
Duration of illness ≤5 days	77 (84.6)	106 (79.7)	1.4 (0.7-2.8)	0.225	1.4 (0.6-3.2)	0.408
Temperature, mean, (SD)	38.0 (1.01)	38.2 (0.94)		0.180		
Fever documented at inclusion	52 (56.5)	89 (66.4)	0.6 (0.4-1.1)	0.086	0.8 (0.4-1.5)	0.472
**Detection of:**						
S. pneumoniae	48 (52.2)	67 (50.0)	1.1 (0.6-1.9)	0.426	1.2 (0.6-2.3)	0.583
Influenza virus	10 (10.9)	18 (13.4)	0.8 (0.3-1.8)	0.359	1.6 (0.6-4.4)	0.335
Parainfluenza virus	15 (16.3)	19 (14.2)	1.2 (0.6-2.5)	0.399	0.6 (0.2-1.5)	0.261
Human adenovirus	13 (14.1)	9 (6.7)	2.3 (0.9-5.6)	0.054	**5.4 (1.9-15.3)**	**0.001**
Human rhinovirus	28 (30.4)	30 (22.4)	1.5 (0.8-2.8)	0.114	1.6 (0.7-3.4)	0.251

* When looking at distribution by age groups, the difference between the proportion of patients with and without severe pneumonia was more important for the age groups below 3-month-old, so for univariate and multivariate analyses we considered the factor “less than 3-month-old”. Some factors were excluded: birth weight that is correlated to low birth weight, duration of illness that is correlated to duration of illness ≤5 days, temperature that is correlated to fever documented at inclusion.

Table S6: Predictive factors for severe pneumonia in ARI patients less than 2 years old.

Factors	Univariate analysis				Multivariable analysis	
	Severe pneumonia	Not severe pneumonia	OR (95% IC)	p-value	OR (95% IC)	p-value
Age groups						
Less than 1-month-old	9 (7.6)	5 (2.8)				
1 to less than 3-month-old	29 (24.66)	13 (7.3)				
3 –month-old to less than 1-year-old	47 (39.8)	74 (41.6)				
1 to less than 2-year-old	33 (28.0)	86 (48.3)				
Age < 3 months*	38 (32.2)	18 (10.1)	**4.2 (2.3-7.9)**	**<0.001**	**6.1 (2.6-14.4)**	**<0.001**
Gender, Male	67 (56.8)	118 (66.3)	0.7 (0.4-1.1)	0.063	0.6 (0.3-1.0)	0.053
Birth weight (gr), Mean (SD)	2975.8 (616.9)	2966.0 (575.1)		0.894		
Low birth weight	15 (14.6)	23 (13.9)	1.1 (0.5-2.1)	0.511	0.9 (0.4-2.2)	0.841
Season (Wet season)	70 (59.3)	132 (74.2)	**0.5 (0.3-0.8)**	**0.005**	0.5 (0.3-1.1)	0.075
PVC 13 received	42 (40.0)	88 (54.7)	**0.6 (0.3-0.9)**	**0.013**	**0.5 (0.3-0.9)**	**0.016**
Duration of illness prior to hospitalization (day), mean (SD)	3.7 (2.1)	4.0 (2.2)		0.177		
Duration of illness ≤5 days	95 (81.2)	142 (80.7)	1.0 (0.6-1.9)	0.519	1.6 (0.7-3.4)	0.525
Temperature, mean, (SD)	37.9 (0.9)	38.0 (0.8)		0.420		
Fever documented at inclusion	65 (55.1)	110 (62.5)	0.7 (0.5-1.2)	0.125	1.1 (0.6-2.1)	0.797
**Detection of:**						
S. pneumoniae	65 (55.1)	97 (54.5)	1.0 (0.6-1.6)	0.508	1.1 (0.6-2.0)	0.858
Respiratory syncytial virus	41 (34.7)	97 (54.5)	**0.4 (0.3-0.7)**	**0.001**	0.8 (0.4-1.6)	0.518
Influenza virus	7 (5.9)	4 (2.2)	2.7 (0.8-9.6)	0.094	2.6 (0.6-11.6)	0.196
Parainfluenza virus	13 (11.0)	17 (9.6)	1.2 (0.5-2.5)	0.412	0.9 (0.4-2.5)	0.906
Human adenovirus	11 (9.3)	8 (4.5)	2.2 (0.9-5.6)	0.079	**5.2 (1.6-17.2)**	**0.007**
Human rhinovirus	27 (22.9)	29 (16.3)	1.5 (0.8-2.7)	0.103	1.5 (0.7-3.3)	0.311

* When looking at distribution by age groups, the difference between the proportion of patients with and without severe pneumonia was more important for the age groups below 3-month-old, so for univariate and multivariate analyses we considered the factor “less than 3-month-old”. Some factors were excluded: birth weight that is correlated to low birth weight, duration of illness that is correlated to duration of illness ≤5 days, temperature that is correlated to fever documented at inclusion.

Table S7: Predictive factors for severe pneumonia in RSV positive patients less than 2 years old.

Factors	Univariate analysis				Multivariable analysis	
	**Severe pneumonia**	**Not severe pneumonia**	**OR (95% IC)**	**p-value**	**OR (95% IC)**	**p-value**
Age groups						
Less than 1-month-old	5 (12.2)	5 (5.2)				
1 to less than 3-month-old	9 (22.0)	7 (7.2)				
3 –month-old to less than 1-year-old	12 (29.3)	44 (45.4)				
1 to less than 2-year-old	15 (36.6)	41 (42.3)				
Age < 3 months*	14 (34.1)	12 (12.4)	**3.7 (1.5-8.9)**	**0.004**	**5.7 (1.5-24.4)**	**0.009**
Gender, Male	20 (48.8)	65 (67.0)	**0.5 (0.2-0.9)**	**0.035**	**0.3 (0.1-0.8)**	**0.020**
Birth weight (gr), Mean (SD)	2930.8 (672.8)	2979.3 (574.4)		0.686		
Low birth weight	4 (11.4)	12 (13.8)	0.8 (0.2-2.7)	0.492	0.8 (0.2-2.9)	0.690
Season (Wet season)	37 (90.2)	90 (92.8)	0.7 (0.2-2.6)	0.422	0.8 (0.2-3.5)	0.720
PVC 13 received	19 (57.6)	44 (51.2)	1.3 (0.6-2.9)	0.337	1.3 (0.5-3.3)	0.607
Duration of illness prior to hospitalization (day), mean (SD)	4.0 (2.0)	4.0 (1.8)		0.841		
Duration of illness ≤5 days	31 (75.6)	80 (83.3)	0.6 (0.3-1.5)	0.205	0.9 (0.3-3.2)	0.885
Temperature, mean, (SD)	38.1 (0.9)	38.0 (0.8)		0.485		
Fever documented at inclusion	24 (58.8)	59 (62.1)	0.9 (0.4-1.9)	0.419	1.4 (0.5-3.9)	0.488
**Detection of:**						
S. pneumoniae	26 (63.4)	60 (61.9)	1.1 (0.5-2.3)	0.510	0.8 (0.3-2.1)	0.633
Influenza virus	0 (0.0)	1 (1.0)	NA	0.703	NA	
Parainfluenza virus	2 (4.9)	1 (1.0)	4.9 (0.4-55.9)	0.210	0.2 (0.01-2.6)	0.218
Human adenovirus	1 (2.4)	4 (4.1)	0.6 (0.06-5.4)	0.533	NA	
Human rhinovirus	3 (7.3)	8 (8.2)	0.9 (0.2-3.5)	0.578	1.7 (0.3-9.1)	0.518

* When looking at distribution by age groups, the difference between the proportion of patients with and without severe pneumonia was more important for the age groups below 3-month-old, so for univariate and multivariate analyses we considered the factor "less than 3-month-old". Some factors were excluded: birth weight that is correlated to low birth weight, duration of illness that is correlated to duration of illness ≤5 days, temperature that is correlated to fever documented at inclusion.

Table S8: Predictive factors for severe pneumonia in RSV negative patients less than 2 years old.

Factors	Univariate analysis				Multivariable analysis	
	**Severe pneumonia**	**Not severe pneumonia**	**OR (95% IC)**	**p-value**	**OR (95% IC)**	**p-value**
Age groups						
Less than 1-month-old	4 (5.2)	0 (0.0)				
1 to less than 3-month-old	20 (26.0)	6 (7.4)				
3 –month-old to less than 1-year-old	35 (45.5)	30 (30.7)				
1 to less than 2-year-old	18 (23.4)	45 (55.6)				
Age < 3 months*	24 (31.2)	6 (7.4)	**5.6 (2.2-14.8)**	**<0.001**	**5.7 (1.6-19.9)**	**0.006**
Gender, Male	47 (61.0)	53 (65.4)	0.8 (0.4-1.6)	0.342	0.7 (0.3-1.6)	0.352
Birth weight (gr), Mean (SD)	2999.7 (589.0)	2951.1 (579.2)		0.617		
Low birth weight	11 (16.2)	11 (14.1)	1.2 (0.5-1.9)	0.452	1.4 (0.4-5.4)	0.632
Season (Wet season)	33 (42.9)	42 (51.9)	0.7 (0.4-1.3)	0.165	0.4 (0.2-1.0)	0.054
PVC 13 received	23 (31.9)	44 (58.7)	**0.3 (0.2-0.7)**	**0.001**	**0.2 (0.08-0.5)**	**0.001**
Duration of illness prior to hospitalization (day), mean (SD)	3.5 (2.1)	4.0 (2.6)		0.195		
Duration of illness ≤5 days	64 (84.2)	62 (77.5)	1.5 (0.7-3.5)	0.195	1.8 (0.6-5.2)	0.295
Temperature, mean, (SD)	37.8 (0.9)	38.1 (0.9)		0.179		
Fever documented at inclusion	41 (53.2)	51 (63.0)	0.7 (0.4-1.3)	0.141	1.1 (0.4-2.7)	0.901
**Detection of:**						
S. pneumoniae	39 (50.6)	37 (45.7)	1.2 (0.7-2.3)	0.321	1.2 (0.5-3.0)	0.623
Influenza virus	7 (9.11)	3 (3.7)	2.6 (0.6-10.4)	0.144	3.0 (0.5-18.4)	0.233
Parainfluenza virus	11 (14.3)	16 (19.8)	0.7 (0.3-1.6)	0.242	1.3 (0.4-3.9)	0.671
Human adenovirus	10 (13.0)	4 (4.9)	2.9 (0.9-9.6)	0.066	**12.3 (2.7-55.3)**	**0.001**
Human rhinovirus	24 (31.2)	21 (25.9)	1.3 (0.6-2.6)	0.290	1.4 (0.5-3.5)	0.528

* When looking at distribution by age groups, the difference between the proportion of patients with and without severe pneumonia was more important for the age groups below 3-month-old, so for univariate and multivariate analyses we considered the factor “less than 3-month-old”. Some factors were excluded: birth weight that is correlated to low birth weight, duration of illness that is correlated to duration of illness ≤5 days, temperature that is correlated to fever documented at inclusion.
